# Predictors of Successful Memory Aging in Older Mexican Adults

**DOI:** 10.1155/2022/9045290

**Published:** 2022-06-25

**Authors:** Rosa Estela García-Chanes, Luis Miguel Gutiérrez-Robledo, Teresa Álvarez-Cisneros, Paloma Roa-Rojas

**Affiliations:** Dirección de Investigación, Instituto Nacional de Geriatría, Mexico City, Mexico

## Abstract

**Background:**

Research suggests a significant association between increasing age and memory impairments. Nevertheless, for some individuals, memory performance stays within or above the normative values of younger subjects. This is known as successful memory aging and is associated with specific neurophysiological features and psychological and lifestyle-related variables. To date, little is known about the association between successful memory aging and intrinsic capacity (IC) defined as “the composite of all the physical and mental (including psychosocial) capacities that an individual can draw on at any point in time” and resilience. Hence, the aim of this study was to determine if longitudinal associations between IC and successful memory aging and resilience exist and to find differences in cognitive performance between Mexican older adults with successful memory aging, older adults with average memory, and older adults with memory impairment.

**Methods:**

Longitudinal data from 590 individuals from the third wave (2012) and the Mex-Cog subsample (2016) of the Mexican Health and Aging Study was analysed. Subjects were classified into 3 groups: (1) older adults with successful memory aging (SUMA), (2) older adults with average memory (AVMA), and (3) older adults with memory impairment (IMA). Cognitive domains of orientation, language, attention, constructional praxis, and executive function were evaluated. IC and resilience were measured using items from the MHAS battery. Analysis of variance and multinomial logistic regressions were used to find differences in IC and resilience across the memory aging groups.

**Results:**

ANOVAs showed significant differences across the three cognitive performance groups in all cognitive domains. Multinomial logistic regression analyses revealed that respondents with higher scores in the psychological and cognitive domains of IC at baseline were more likely to have successful memory aging in the subsequent wave of the study. More resilient subjects in 2012 were not more likely to become a SUMA in 2016. However, this could be a result of the way resilience was measured.

**Conclusion:**

Our main findings suggest that intrinsic capacity could be used as a predictor of successful memory aging specifically in the psychological and the cognitive domains. More longitudinal studies are needed to further examine these associations.

## 1. Introduction

Memory aging is highly heterogeneous. Research focusing on healthy adults suggests a significant association between increasing age and memory impairments [1]; however, a selected group of adults show memory performance within or above the normative values for younger subjects [2]. Adults having this kind of performance are typically called superagers or (less frequently) successful memory agers [3]. For example, the Northwestern University SuperAging Study [4] classified adults aged 80 years or more, with performance on a delayed recall task at or above the normative values for individuals aged 56 to 66, as superagers. Explicative variables for this heterogeneity are still under study.

Evidence suggests that superagers or successful memory agers have distinct neurophysiological features such as a higher cortical thickness of specific structures like the hippocampus, anterior temporal cortex, rostral medial prefrontal cortex, and anterior cingulate cortex [5–9] and differences in cerebral atrophy rates [10]. In addition to neurophysiological features, evidence points to an association with other psychological and lifestyle variables such as extroversion. Superagers report more positive social relationships compared to average older adults [11, 12]. Superagers also report higher rates of physical activity [13–16].

Another variable related to successful memory aging is resilience [17]. Despite its many definitions, resilience was defined as doing well in face of stressful events because of protective personal resources [18] such as a positive outlook and emotional regulation [19]. Evidence suggests that resilience positively impacts exceptional longevity [20] and cognition. Superagers show stable cognitive performance through time in memory and nonmemory domains [21, 22], which is why they are also called resilient agers.

However, none of these measures reflects the global capacities of the individual. Considering that in 2015, the World Health Organization (WHO) redefined the concept of “healthy aging” changing the focus from the presence or absence of disease to a functioning-based approach where “functional ability” is determined by the combination of the intrinsic capacity (IC) of the individual, relevant environmental characteristics, and the interactions between the individual and these characteristics [23].

IC is defined as “the composite of all the physical and mental (including psychosocial) capacities that an individual can draw on at any point in time” [24]. It has a wide distribution across the life course. IC gradually declines with increasing age; however, there are some exceptional individuals aged 80 years or over able to maintain an IC higher than younger adults [25]. Evidence suggests that IC provides predictive information about subsequent functioning [26], even after considering the effects of multimorbidity [27].

Current estimates of IC consider five distinct domains: locomotor, cognitive, psychological, sensory, and vitality [28]. Deficits in any of these domains are considered declines in IC. Multiple conditions, including cognitive impairment, have been associated with declines in IC [29]. Associations between the decline of IC and frailty and other chronic conditions have also been found in the Mexican population [30, 31]. A recent study by the 10/66 Dementia Research Group showed that 12.5% of the subjects with a decline in IC had dementia, while only 0.4% of subjects without IC declines presented this condition [32]. However, the effects of IC decline on cognition are still largely unknown. As older adults from Mexico have a high prevalence of risk factors for dementia and memory impairment such as hypertension, obesity, diabetes, and depression, this is an interesting group to study these associations. This is also relevant because of the little evidence from middle-income countries like Mexico [33, 34].

The study main objective was to find whether a longitudinal relationship between IC and successful memory aging existed in older Mexican adults. It is first aimed at determining the differences in cognitive performance between successful memory agers, average memory agers, and adults with memory impairment. Second, it examined whether respondents with better resilience and IC scores in 2012 were more likely to belong to the successful memory aging group in the 2016 wave of the Mexican Health and Aging Study (MHAS) [35].

## 2. Materials and Methods

### 2.1. Subjects

**Figure 1:**
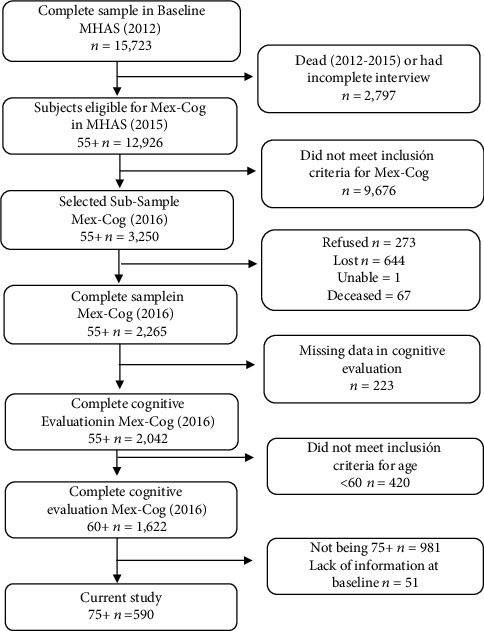
Flow chart of study participants.

Longitudinal data from the MHAS (https://www.MHASweb.org) third wave (2012) was used as a baseline, and data from the Mex-Cog subsample (2016) was used as the follow-up. The MHAS (https://www.MHASweb.org) study began in 2001, with a representative sample of adults 50 years of age and over from urban and rural areas of Mexico. It was designed to prospectively evaluate the impact of disease in older adults from Mexico [35, 36] and currently has five rounds (2001, 2003, 2012, 2015, and 2018). In 2016, the Mex-Cog, a subsample of 2,265 participants, was designed to be part of the Harmonized Cognitive Assessment Protocol (HCAP), allowing cross-national comparisons of the worldwide prevalence and trends of dementia in aging populations [37]. Subjects in the Mex-Cog sample were selected from the fourth wave of MHAS (2015). The inclusion criteria for Mex-Cog included having 55 or more years of age and a complete direct interview in 2015. However, individuals from only 8 states were selected using stratified sampling procedures. A total of 3,250 eligible subjects were included, but interviews were completed for 2,265 subjects [38]. For the present study, a total of 590 subjects from the Mex-Cog subsample were selected. The exclusion criteria included not being 75 years old and over and the lack of information in 2012 (see Figure 1).

### 2.2. Successful Memory Aging

All 590 subjects were classified into three groups: successful memory agers (SUMAs), average memory agers (AVMAs), and adults with memory impairment (IMA). For classification, the normative values of the “10-word learning test” from the CERAD protocol, included in the Mex-Cog battery, were used [39]. Subjects aged over 75 years and with memory performance within or above the normative values for subjects aged 65 to 69 years were considered successful memory agers, hence included in the SUMA group. Subjects aged 75 and over, with memory performance within average normative values, were included in the AVMA group. Finally, subjects aged 75 or more and with a memory performance below normative values were included in the IMA group.

### 2.3. Cognition

Five domains proposed by the Mex-Cog project [38]: orientation, attention, language, constructional praxis, and executive function, were used to assess cognition. The orientation task required subjects to answer questions about the context (day of the month, month, year, day of the week, What time is it? Where are we now? How can I get to a store?, country, and state). Results were scored 0-9. The attention task required subjects to do visual detection and countdown. Results were scored in the range 0-65. The language tasks required subjects to follow instructions, name objects, repeat, and read and write a sentence. Results were scored 0-14. The constructional section required subjects to copy 4 figures scoring respondents 0-12 points. Finally, the executive function tasks required subjects to do serial subtraction of 3, serial subtraction of 7, verbal fluency, symbols and digits, similarities, and “go not go,” scoring participants 0-83 points.

### 2.4. Intrinsic Capacity

According to the Integrated Care for Older People (ICOPE) guidelines [40], IC comprises five domains: cognition, psychological, senses (vision and hearing), vitality, and mobility. Variables from the corresponding sets of MHAS questionnaires third wave (2012) were selected. Regarding cognition, the verbal recall memory and orientation tests were used assigning a value of 1 which was assigned to respondents with deficits in any of these. For the psychological domain, two questions from the MHAS depressive symptom questionnaire were used: “During the past week, have you felt depressed?” and “During the past week, was everything you did difficult to do?.” A value of 1 was assigned to subjects who had at least one affirmative answer. Self-rated vision and hearing were assessed using the questions “How is your vision (with or without glasses)?” and “How is your hearing/auditory range (using hearing aid or auditory device)?.” A value of 1 was assigned when the answers included fair, poor, or legally blind and fair, poor, or legally deaf, respectively. Regarding vitality, subjects who answered “yes” to “Compared with two years ago, did you lose 5 kilograms or more?” or “In the last two years, have you eaten less because of loss of appetite, digestive problems, and difficulties chewing or swallowing?” received a value of 1. For mobility, a value of 1 was assigned if the individual answered “yes” to any of the following: “Do you have difficulty walking one block?” or “Do you have difficulty climbing several flights of stairs without resting?” [41, 42]. Finally, scores were added to have one single score, ranging from 0 to 6.

### 2.5. Resilience

The MHAS does not include a specific instrument for measuring resilience; hence, a resilience measure was developed considering that in the face of stressful events, resilient subjects do well because of protective internal resources [18] such as positive outlook and emotional regulation [19]. Our measure is based on a previous longitudinal study on multidimensional resilience in Mexican older adults [43]. However, for our study, we approached resilience without considering its development over time. Hence, first, we identified common stressors for older adults which include a significant loss or a serious fall (fracture) or illness. Significant loss was defined as having experienced recent widowhood or the death of a sibling, and it was operationalized giving 1 point to those experiencing this loss and 0 to those who had not. One point was added to those experiencing serious falls (fractures) and to those recently diagnosed with an illness and 0 to those who had not. Second, we identified two personal resources, positive outlook and emotional regulation. Positive outlook or remaining hopeful regardless of stressful events was operationalized using two items: (a) self-rated health, where 1 was assigned to those who rated their health as excellent, very good, or good and 0 to those who rated their health as fair or poor and (b) life satisfaction using the affirmation “The conditions of my life are excellent,” where 1 was assigned to respondents who agreed and 0 to those who did not. Emotional regulation is related to internal locus of control; this was operationalized with one item measuring internal locus of control. The item was “do you think you can improve your health?”; respondents providing an affirmative answer were coded 1 while those answering no were coded 0. Third, subjects were categorized into three groups: subjects having zero stressors and zero personal resources were included in the not resilient group (NRES); subjects having one or more stressors and two or three personal resources were included in the resilient subjects group (RES); finally, subjects having zero stressors and two or three personal resources were included in the less resilient group (LRES).

### 2.6. Covariables

Sociodemographic variables included age, sex, marital status (married/consencual union, single, widowed, and divorced), educational achievements, socioeconomic level, characteristics of childhood household (having toilet inside the house before the age of ten), and having a retirement pension. Health-related variables included self-reported diabetes and hypertension. Other health conditions such as heart attack, heart disease, lung disease (asthma or emphysema), cancer, arthritis, and stroke were considered one variable (having one or more). In order to include lifestyle, a variable for social and recreational activity was created. This variable considered taking care of older people, taking care of children, voluntary work, training, attending sports, reading, playing board games, talking to people, and doing crafts. The value of 1 was given to those who reported two or more activities.

### 2.7. Statistical Analyses

In order to find whether respondents in each of the memory groups differed with regard to cognitive performance (the first aim of the study), a series of one-way ANOVAs were performed, where the group was included as a between-subject factor (SUMA, AVMA, and IMA) and cognitive performance (orientation, attention, language, constructional praxis, and executive function) as a dependent variable. Adjusted *p* values for multiple comparisons at 0.001 were used. Then, post hoc tests (Tukey's honestly significant difference method) were used for pair-wise comparisons.

To address whether respondents with better resilience and intrinsic capacity scores in 2012 were more likely to report successful memory aging in 2016 (the second aim of the study), a multinomial logistic regression analysis having successful memory aging as the outcome variable and IC, resilience, and significant covariables from baseline (2012) as predictors was performed. Odds ratios and 95% confidence intervals were calculated, and all statistical analyses were carried out using STATA 14.

## 3. Results and Discussion

As shown in Table 1, in 2012, significant differences were seen in the cognitive, psychological, and vision domains of IC. SUMAs reported the lowest prevalence of affections in the global IC score (35.7%) with a lower proportion reporting cognitive (33.3%), psychological (31.9%), and visual (33.3%) impairments when compared to AVMAs and IMAs. However, they reported the highest prevalence of mobility (57.1%) impairments. When looking into resilience, results also showed significant differences between the three groups. As expected, a higher proportion of SUMAs fitted into the resilient subjects group (37.8%). This table also shows that the mean age of respondents was 76.6 years; 55.6% were women and had an average of 3.8 years of formal education. Significant differences between the three groups were seen between groups. The oldest group was the IMA group, with a mean age of 77.6 while the group with a significantly higher percentage of women (66.2%) was the SUMA group. The AVMAs had a higher percentage of married subjects (55.9%). SUMAs had the highest percentage of subjects with a toilet inside their house before the age of 10 (37.8%). This group also reported the highest average of formal years of education (6.4 years) and the highest amount of income (40.5%), and a higher percentage of subjects received a pension (44.6%) when compared to AVMAs and IMAs. Finally, a higher proportion of SUMAs referred to having the diagnosis of hypertension (60.8%), and a lower proportion reported having diabetes (21.9%) when compared to the other cognition groups.

Results from Table 2 show the mean scores and standard deviations of each group for the five cognitive tasks. SUMAs outperformed AVMAS and IMAs in all cognitive domains. Post hoc analyses revealed significant differences in orientation (*F* = 30.1, *p* < 0.0001) and executive function (*F* = 43.7, *p* < 0.0001) between the three groups. Second, SUMAs also outperformed IMAs in attention, language, and constructional praxis tasks. Post hoc analyses revealed significant differences between attention (*F* = 24.5, *p* < 0.0001) language (*F* = 26.3, *p* < 0.0001), and constructional praxis (*F* = 13.19, *p* < 0.0001) tasks. Post hoc results are shown in Table 3.

Finally, results from the multinomial logistic regressions looking into whether IC, resilience, and other covariates from 2012 predict successful memory aging in 2016 are presented in Table 4. Respondents reporting no affections in the psychological and cognitive domains in 2012 were more likely to become a SUMA in 2016. However, the OR reporting mobility impairments in 2012 was higher for this group when compared to IMAs but not AVMAs.

Respondents reporting affections in the IC psychological (OR = 2.37; IC (1.28-4.44)) and cognitive (OR =2.01; IC (1.10-3.66)) domains in 2012 were more likely to become an IMA in 2016, while those reporting mobility impairments had a significantly lower likelihood (OR = 0.48; IC (0.26-0.89)) of belonging to this group. Similarly, the odds of becoming an AVMA in 2016 were higher among those reporting affections in the psychological (OR = 1.87; IC (0.96-3.62)) domain of IC in 2012.

Individuals from the more resilient group in 2012 were not more likely to become a SUMA in 2016; however, neither were those belonging to the less resilient group. Finally, when looking into the other covariates, results also suggest that individuals with higher age in 2012 were more likely to become SUMA in 2016 than an AVMA (OR = 0.85; IC (0.78-0.85)). Similarly, respondents with a higher number of years in formal education were also more likely to become a SUMA than an AVMA or an IMA in 2016. The effect of the other covariates from 2012 did not seem to affect the memory aging group in 2016.

### 3.1. Discussion

The current study had two aims. First is to determine whether cognitive performance differed significantly across the three memory aging groups: SUMAs, AVMAs, and IMAs cross-sectionally. Second is to examine the longitudinal association between IC and resilience and successful memory aging using data from the Mexican Health and Aging Study (MHAS) waves 2012 and 2016 [35].

Results from comparisons in cognitive performance (orientation, attention, language, constructional praxis, and executive function) tasks between groups suggest subtle differences between all groups, aside from the memory domain. Furthermore, when looking into differences between SUMAs and IMAs, SUMAs showed higher cognitive performance across all domains. However, SUMAS outperformed AVMAs only in the orientation and executive function domains.

As expected, the best cognitive performance overall was found in SUMAs. This is consistent with findings from other studies suggesting a significantly higher cognitive performance of SUMAs outside the memory domain [13]. This could be explained by the different patterns of cognitive changes seen in healthy older adults. As it could be assumed that both SUMAs and AVMAs do not have cognitive impairments, differences in other domains aside from memory could be explained by the different patterns of cognitive changes [44].

To find whether individuals with better resilience and intrinsic capacity in 2012 were more likely to become a SUMA in 2016, multinomial logistic regression analyses were performed. Results showed that respondents with psychological or cognitive affections at baseline were more likely to become an IMA or an AVMA in 2016, thus less likely to become a SUMA. As briefly described in Materials and Methods, the psychological domain was measured using items from the depressive symptom scale, and evidence suggests that successful memory aging is negatively associated with depression [45]. Affections in the cognitive domains were also significantly related to a lower likelihood of belonging to the SUMA group in 2016. This is in line with previous studies suggesting that SUMAs show stable cognitive performance through time in all cognitive domains [21].

With regard to the effect of resilience, the SUMA group showed the highest prevalence of resilient subjects as expected; however, our findings did not show that more resilient individuals in 2012 were more likely to become a SUMA in 2016. Possible explanations for this include a measurement bias of resilience and the negative association of resilience with physical health. For the current study, resilience was measured considering that in the face of stressful events, resilient subjects do well because of protective personal resources. However, evidence suggests there is a multidimensional nature of resilience [19, 43], and the only domain considered for the operationalization of resilience in this study was the individual dimension. Perhaps, the dimensions not considered for this measure significantly impact memory. With regard to the negative effects of resilience on physical health, some studies have found an association between resilience and hypertension, particularly in men from lower-income categories [46, 47]. The SUMA group showed the highest prevalence of hypertension.

Other variables associated with a higher likelihood of belonging to the SUMA group in 2016 were age and years of formal education. The protective effect of schooling on cognition has been widely recognized [48, 49], and evidence suggests that this relationship is a result of the increased cerebral volume and metabolism seen among individuals with higher educational achievements [50].

Some of the strengths of this study when compared to previous research are the large sample size and the inclusion of multiple variables. Also, to our knowledge, this is the first study including Latin-American individuals. However, some limitations must be mentioned. First, measurements of neurophysiological status are lacking, and despite having related information, the associations between brain pathology and psychological and lifestyle variables could not be controlled for. Second, subjects were classified according to Mexican norms; however, there are normative values only looking to memory tasks; hence, the prevalence of impairments in other cognitive domains was disregarded. In order to understand the cognitive functioning in SUMAs, analysing performance across all cognitive domains would have been more suitable. Third, attrition of the most vulnerable should also be considered when looking into differences between SUMAS, AVMAs, and IMAs, as it is possible that a higher proportion of IMAs abandoned the study. Future research should explore other dimensions of resilience and how these other dimensions associate with cognition in general. Considering the increasing proportion of older adults, the understanding of factors associated with SUMA is crucial for the development of public policies aimed at fostering SUMA.

## 4. Conclusions

In conclusion, results from this study show that SUMAs and IMAs differed across all cognitive domains. This was not true for SUMAs and AVMAs, as significant differences are seen only in the memory, orientation, and executive function domains. Psychological and cognitive domains of IC were able to predict a higher likelihood of becoming a SUMA. A higher percent of resilient subjects in 2012 belonged to the SUMA group in 2016, but there were no significant differences between groups. Altogether, these findings point to the importance of psychological and cognitive factors for achieving successful memory aging. Policies focusing on mental health and better cognitive aging should be put in place in order to promote successful memory aging in the population.

## Figures and Tables

**Table 1:** Characteristics of the study sample from baseline (2012) (*n* = 590).

Variables (wave 2012)	Total	SUMA	AVMA	IMA	Sig.
*n*	590	74	170	346	
Age					
Mean (sd)	76.6 (5.4)	76.6 (3.6)	75.8 (4.8)	77.6 (5.8)	∗∗∗
[Min–Max]	[70-102]	[72-93]	[70-94]	[70-102]	
Sex (%)					
Women	55.6	66.2	53.5	54.3	
Marital status (%)					
Married/consensual union	53.7	47.3	55.9	53.9	
Childhood household (%)					
Yes	25.8	37.8	29.6	21.4	∗∗∗
Years of formal education					
Mean (sd)	3.8 (4.0)	6.4 (4.9)	4.0 (3.9)	3.2 (3.6)	∗∗∗
Socioeconomic level^1^ (%)					
Without income -3° quintile					
4°-5° quintile	25.0	40.5	24.1	22.0	∗∗∗
Pension (%)					
Yes	32.7	44.6	35.9	28.7	∗∗∗
Intrinsic capacity (%)					
3-6 domains affected	43.0	35.7	43.4	44.7	
IC affected domain (%)					
Psychological	47.3	31.9	46.6	51.1	∗∗∗
Cognition	50.6	33.3	46.6	56.7	∗∗∗
Vision	48.3	33.3	52.8	49.4	∗∗∗
Audition	1.2	0.0	1.2	1.5	
Mobility	49.9	57.1	49.4	48.5	
Vitality	35.1	41.7	36.8	32.8	
Hypertension (%)					
Yes	52.9	60.8	57.1	49.1	
Diabetes (%)					
Yes	22.9	21.9	26.5	21.3	
Other health conditions^2^ (%)					
1+	32.1	35.1	28.8	33.1	
Recreational and social activities^3^ (%)					
2 or more activities	40.2	59.5	42.4	35.0	∗∗∗
Resilience^4^ (%)					
NRES	24.8	17.6	22.4	27.5	∗∗∗
RES	28.3	37.8	22.4	29.2	
LRES	47.0	44.6	55.3	43.4	

^1^Individual earned income, pension income, transfer income, business income, or property rent income. ^2^Having one or more health conditions including cancer, lung disease, heart attack, stroke, and arthritis. ^3^Taking care of older people, taking care of children, voluntary work, training, attending sports, reading, playing board games, talking to people, and doing crafts. ^4^Not resilient group (NRES), resilient subjects group (RES), and less resilient group (LRES). Chi-squared test: ^∗∗∗^*p* < 0.05; ^∗∗^*p* < 0.10. ANOVA test: ^∗∗∗^*p* < 0.05; ^∗∗^*p* < 0.10.

**Table 2:** Group comparisons on cognitive performance (*n* = 590).

Cognition	Total	SUMA	AVMA	IMA	*F* (df)
Orientation	5.8 (2.2)	7.3 (1.7)	6.2 (1.9)	5.3 (2.3)	30.19^∗∗∗^
Attention	18.8 (15.5)	28.2 (15.0)	21.2 (15.2)	15.5 (14.8)	24.59^∗∗∗^
Language	11.3 (1.5)	12.8 (1.5)	11.9 (2.1)	10.7 (2.9)	26.31^∗∗∗^
Constructional praxis	7.1 (2.5)	8.4 (2.3)	7.3 (2.4)	6.7 (2.6)	13.19^∗∗∗^
Executive function	22.9 (16.1)	36.5 (15.8)	25.0 (13.9)	18.9 (15.5)	43.75^∗∗∗^

^1^ANOVA *F*-test mean (sd); ^∗∗∗^Sig < 0.001.

**Table 3:** Post hoc comparisons using Tukey's HDS. Mean differences shown.

	Cognition	GROUPS		
		SUMA	AVMA	IMA
SUMA	Orientation	1	1.078^∗^	1.977^∗^
	Attention		6.955	12.629^∗^
	Language		0.927	2.083^∗^
	Constructional praxis		1.102	1.685^∗^
	Executive function		11.436∗	17.566^∗^
AVMA	Orientation		1	0.899^∗^
	Attention			5.674^∗^
	Language			1.157^∗^
	Constructional praxis			0.583
	Executive function			6.130^∗^
IMA	Orientation			1
	Attention			
	Language			
	Constructional praxis			
	Executive function			

^∗^Sig.<0.001.

**Table 4:** Multinomial logistic regressions predicting successful memory aging (*n* = 590).

Variables (2012)	SUMA (*n* = 74)			
	IMA (*n* = 346)		AVMA (*n* = 170)	
	RRR (95% IC)	Sig.	RRR (95% IC)	Sig.
Sex				
Men (ref.)				
Women	0.58 (0.29-1.14)	0.112	0.50 (0.24-1.02)	0.057
Age (2012)	0.99 (0.94-1.05)	0.842	0.85 (0.85-0.97)	0.003
Marital status				
Union	0.88 (0.46-1.70)	0.71	0.70 (0.35-1.39)	0.304
Childhood household				
No (ref.)				
Yes	0.99 (0.50-1.96)	0.982	1.35 (0.66-2.75)	0.416
Education years	0.89 (0.82-0.96)	0.003	0.90 (0.83-0.98)	0.011
Income quintile^1^				
4°-5° quintile	0.75 (0.39-1.44)	0.386	0.64 (0.32-1.29)	0.216
Recreational and social activities^2^	0.66 (0.36-1.24)	0.200	0.69 (0.35-1.34)	0.217
Domain affected				
Psychological	2.37 (1.27-4.44)	0.007	1.87 (0.96-3.62)	0.066
Cognition	2.01 (1.10-3.66)	0.023	1.60 (0.84-3.03)	0.153
Mobility	0.48 (0.26-0.89)	0.02	0.60 (0.31-1.15)	0.121
Hypertension				
Yes	0.81 (0.44-1.47)	0.486	1.03 (0.54-1.97)	0.917
Resilients^3^				
NRES (ref.)				
RES	0.95 (0.40-2.27)	0.915	0.62 (0.25-1.58)	0.321
LRES	0.92 (0.40-2.13)	0.843	1.02 (0.42-2.46)	0.962

^1^Individual earned income, pension income, transfer income, business income, or property rent income. ^2^Taking care of older people, taking care of children, voluntary work, training, attending sports, reading, playing board games, talking to people, and doing crafts. ^3^Not resilient subjects group (NRES), resilient subjects group (RES), and less resilient subjects group (LRES).

## Data Availability

The quantitative data supporting this study comes from the MHAS (Mexican Health and Aging Study) dataset. Data files and documentation are for public use and available at https://www.MHASweb.org. This has been cited in Materials and Methods of the study. The processed data are available from the corresponding author upon request.
